# Effects of Cortisol on the Intestinal Mucosal Immune Response during Cohabitant Challenge with IPNV in Atlantic Salmon (*Salmo salar*)

**DOI:** 10.1371/journal.pone.0094288

**Published:** 2014-05-08

**Authors:** Lars Niklasson, Henrik Sundh, Rolf-Erik Olsen, Fredrik Jutfelt, Karsten Skjødt, Tom O. Nilsen, Kristina Snuttan Sundell

**Affiliations:** 1 Fish Endocrinology Laboratory, Department of Biological and Environmental Sciences, University of Gothenburg, Gothenburg, Sweden; 2 Department of Animal Welfare, Institute of Marine Research, Matredal, Norway; 3 Department of Cancer and Inflammation, University of Southern Denmark, Odense, Denmark; 4 Department of Biology, University of Bergen, Bergen, Norway; INRA, France

## Abstract

Infectious pancreatic necrosis virus (IPNV) causes high incidence of disease in salmonids during the first period after SW transfer. During this period as well as during periods of stress, cortisol levels increase and indications of a relationship between IPNV susceptibility and cortisol have been suggested. The intestine is an entry route and a target tissue for IPNV displaying severe enteritis and sloughing of the mucosa in infected fish. The mechanisms behind effects of the virus on the intestinal tissue and the impact of cortisol on the effect remain unclear. In the present study, Atlantic salmon post smolts treated with or without slow release cortisol implants were subjected to a cohabitant IPNV challenge. Analysis of genes and proteins related to the innate and acquired immune responses against virus was performed 6 days post-challenge using qPCR and immunohistochemistry. An increased mRNA expression of anti-viral cytokine interferon type I was observed in the proximal intestine and head kidney as a response to the viral challenge and this effect was suppressed by cortisol. No effect was seen in the distal intestine. T-cell marker CD3 as well as MHC-I in both intestinal regions and in the head kidney was down regulated at the mRNA level. Number of CD8α lymphocytes decreased in the proximal intestine in response to cortisol. On the other hand, mRNA expression of Mx and IL-1β increased in the proximal intestine and head kidney in IPNV challenged fish in the presence of cortisol suggesting that the immune activation shifts in timing and response pathway during simulated stress. The present study clearly demonstrates that IPNV infection results in a differentiated epithelial immune response in the different intestinal regions of the Atlantic salmon. It also reveals that the epithelial immune response differs from the systemic, but that both are modulated by the stress hormone cortisol.

## Introduction

The parr-smolt transformation and the subsequent sea water (SW) migration are important developmental life stage transitions for Atlantic salmon during which changes in the endocrine as well as the immune system occurs [1]–[3]. The changes in physiology, anatomy and behavior in order to prepare the fish for a life in SW are extensively studied, whereas the concomitant changes occurring in the immune system are less well described [4]–[7]. Both up- and down-regulation of different innate immune markers during smoltification and SW transfer have been reported [8], [9]. An up-regulation of immune functions has been accounted stimulatory effects of thyroid hormones and growth hormone and down-regulation are suggested to be caused by increased cortisol levels during these periods [10]–[13]. However, studies as well as experience from aquaculture show that there is a window of about 3 months, starting directly following sea water transfer, with increased susceptibility to pathogens [14].

Cortisol is one of the main developmental hormones, promoting hypo-osmoregulatory ability, with a peak during the parr-smolt transformation and a further increase after transfer to SW [15]. Cortisol is also one of the major stress hormones resulting in several secondary and tertiary stress responses [16]. Among the secondary stress responses, a reduced intestinal integrity has been proposed as a consistent indicator of both acute and chronic stress [17]–[19].

Stress is well known to affect the immune system of fish both systemically and locally, at the intestinal level [13], [20]–[22]. In the salmon intestine, a down-regulated mRNA expression of IL-1β, IFNγ and an alteration of IL-10 together with an increased infiltration of neutrophils have been observed as responses to various stressors [22]. Stress has therefore been suggested to alter the salmonid immune response to pathogens [20].

One of the main problems for salmonid aquaculture is the window of increased susceptibility to infections, e.g. infectious pancreatic necrosis virus (IPNV), 1-4 weeks after SW transfer [23]. Much effort has been put into investigating the physiological and immunological mechanisms that control infection and protection against the virus [24]–[27]. IPNV is a double stranded RNA virus belonging to the Aquabirnaviruses of the *Birnaviridae* family and infection can lead to high mortality and turn surviving fish into lifelong carriers of virus [28]. There are several reference types of the virus, e.g. VR299, Sp and Ab isolated from disease outbreaks [29]. In Norway, the Sp serotype is commonly used in viral challenge experiments [24]–[26]. IPN disease leads to necrosis of pancreatic acinar cells, liver and kidney [28]. One of the first target tissues for IPNV is the intestine, where acute enteritis, sloughing of mucosa and increased paracellular permeability are commonly observed [30]–[32]. These early effects of IPNV infection in the gastrointestinal tract together with the positive demonstration of translocated virus across the intestine suggests this tissue to be a major entry route for the virus [31].

The diverse anti-viral response includes secretion of interferon type I (IFN type I) and II (IFNγ) which activate intracellular signaling pathways, in turn resulting in diverse cellular responses [33]. In salmonid cells, recombinant IFN increases mRNA expression levels of STAT1, of the JAK/STAT pathway, followed by an increased expression of antiviral protein Mx [34]. One of the evasive mechanisms of IPNV is to inhibit Mx expression in the host and thereby dampening the effect of STAT1 phosphorylation [33], [34]. The anti-viral defense further includes activation of T cells in the specific cellular immune response and involves antigen presentation via the MHC-I complex to CD8 positive T cells [25].

Stress has recently been reported to have marked effects on anti-viral processes in Atlantic salmon. Repeated stress in combination with immersion exposure to IPNV resulted in down-regulation of IFN type I and an increased replication of IPNV [35]. Also, long-term hypoxia induced stress changed the timing of the intestinal epithelial immune response towards synthetic double stranded RNA, poly I:C [20].

Therefore, the aim of the present study was to investigate the effects of IPNV infection on the local intestinal anti-viral response in Atlantic salmon and to assess the effect of high circulating levels of cortisol, as a mimic of stress, on this response.

The intestinal mucosal anti-viral pathways were up-regulated in response to a cohabitant challenge with IPNV of the Sp strain and cortisol was shown to suppress these effects. Early immune markers as well as immune cell markers, mortality and IPNV prevalence was monitored and are discussed in relation to IPNV infection and cortisol treatment.

## Materials and Methods

### Fish

275 Atlantic salmon, hatched in mid-January, were kept in fresh water (FW) at approximately 10 °C under continuous light (24L), from first feeding in late February, to reach parr stage (in late August) at the Institute of Marine Research (IMR) in Matre (61 °N), Norway. Thereafter, the fish were subjected to a transient change in photoperiod to 12L:12D for 6 weeks followed by 6 weeks of 24L:0D to produce out-of-season smolts [36]. After this period the smolts, weighing 108±3 g, were transported to IMRs disease challenge laboratory and randomly divided up into eight 150 L flow through FW tanks (8.5 L min^−1^ maintaining O_2_ at 9.2±0.2 mg L^−1^ at a water exchange rate of 3.3 times h^−1^, temperature: 8.9±0.2 °C). The fish were allowed to acclimatize to the new tanks for 3 weeks before initialization of the experiment and were fed a commercial diet (Biomar 50 in FW and Skretting 3 mm) ad libitum, throughout the acclimation and the experimental periods.

The experiment was initialized by a gradual exposure to SW by changing the inlet from FW to SW (8.5 L min^−1^ maintaining O_2_ at 9.2±0.2 mg L^−1^ and a water exchange rate of 3.3 times h^−1^) over a six hour period (unfiltered 9.3±0.1 °C SW from 120 m depth, salinity of 34.5‰). Thereafter, the fish were left undisturbed for 10 days before implantation of cortisol (in mid-November). All fish were treated according to the Norwegian national legislation for laboratory animals throughout the experiment. The experiment was approved by Forsøksdyrutvalgets tilsyns- og søknadssystem (FOTS), Mattilsynet, Forsøksdyrutvalget, Brumunddal, Norway as stated in ethical permit 08/48706. The experiment was carried out using a minimum critical mass of animals to avoid hierarchy formation or over-crowding. The animals and water conditions were under daily surveillance throughout the experiment. Injected fish were allowed to recover for 24 hours before their status was inspected. Any animal showing signs of abnormal behavior, reduced swimming capacity or with visible injuries were netted, anesthetized and euthanized. During the recovery period of 10 days after SW exposure and before cortisol implantation, there was a mortality of six fish randomly distributed over the 8 tanks. After cortisol or vehicle implantation one fish in one of the cortisol implanted groups diseased.

### Cortisol implants

Ten days after SW introduction, all fish from 6 of the 8 tanks were randomly netted, anesthetized in metomidate (∼6 mg L^−1^) and intraperitoneally (i.p.) implanted with a slow release implant with or without cortisol (+F/-F) and returned to their respective tank (n = 36, density 22 kg m^−3^). The remaining two tanks were maintained as non-treated controls throughout the experiment.

Fish in the +F group received cortisol (Sigma-Aldrich Sweden AB, Stockholm, Sweden), at a dose of 50 µg g^−1^ body weight (BW) dissolved in vegetable oil:vegetable shortening 1∶1, at a volume of 5 µL g^−1^ BW and the -F group received the implant without cortisol at the same volume. The cortisol implants have been shown to result in increased circulating levels of cortisol in the fish up to one month [37].

In the present study the cortisol implants resulted in a significantly increased plasma level of cortisol in the +F group compared to –F and non-challenged controls as reported previously [31]. In the +F group the average level of cortisol was 319.2 ± 46.9 ng mL^-1^, in the –F group 9.7±2.9 ng mL^-1^ and in the non-implanted control 6.7±1.9 ng mL^-1^, respectively.

### Cohabitant IPNV challenge

One week after implantation, the cohabitant challenge was started (day 0). Six implanted fish (+F/-F) from each tank were randomly netted, anesthetized in metomidate (∼6 mg L^−1^) and i.p. injected with a viral dose of 1 × 10^7^ TCID_50_ (200 µL) of IPN virus, of the Sp strain, and re-introduced to their respective tanks [29], [31]. The injected fish functioned to challenge the remaining fish in the tank as cohabitants and were adipose-fin clipped to distinguish them from the non-injected fish. This experimental design resulted in 3 treatment groups, one infected with IPNV and implanted with cortisol (+IPNV+F), one infected with IPNV and implanted with vehicle (+IPNV-F) and one non-challenged and non-implanted control (-IPNV). Mortality of non-injected fish was monitored daily in the +IPNV+F and +IPNV-F groups from start of the IPNV challenge up to 41 days post-challenge.

### Sampling

After 6 days of cohabitant exposure (day 12), head kidney and intestinal samples were taken for a total of 9 fish from each group (3 fish per tank of the implanted fish and alternating 4 or 5 fish per non-implanted control tank). Fish were euthanized with a lethal dose of metomidate (∼12.5 mg L^−1^) and followed by a sharp blow to the head. Head kidney samples were placed in liquid nitrogen for later mRNA extraction. The intestine was divided into a proximal and a distal part and a small circular portion of each part was cut out and placed in Hollande’s fixative for 24 h at 4°C, washed and transferred to 70% EtOH and stored for immunohistochemistry analysis. The remaining intestinal parts were cut longitudinally and the mucosa was scraped of and placed in liquid nitrogen for quantitative PCR (qPCR) analysis. A final sampling of proximal intestine and head kidney from +IPNV+F and +IPNV-F fish was performed at day 28 to examine degree of infection by qPCR analysis.

### qPCR

The mRNA expression of immune markers was assessed using qPCR. Tissue samples were homogenized using a Precellys homogenizer (Bertin, France). Approximately 30 mg of each sample were placed in tubes with TRIzol (Invitrogen/Life Technologies Ltd, Paisley, UK) and ceramic beads, Lysing Matrix D (Q-Biogene/MP Biomedicals, Illkirch, France). The RNA was extracted using an iPrep Purification Instrument (Invitrogen/Life Technologies Ltd, Paisley, UK). The RNA quality of randomly selected samples was analyzed in the Bioanalyzer system and the RNA was quantified using a Nanodrop (Thermo, Scientific, Wilmington, USA) and diluted to obtain 2000 ng total RNA per sample. cDNA was synthesized using the Invitrogen SuperScript III First-Strand Synthesis System (Life technologies Ltd, Paisley, UK) with random primers in 20 µl final reaction volume.

The qPCR assays used are listed in Table 1. Primers and hydrolysis probes (FAM-MGBNFQ; Applied Biosystems/Life Technologies Ltd, Paisley, UK) were verified using NCBIs BLAST tool (NCBI, Bethesda, USA) and efficiencies tested in the Bio-Rad iQ5 Real-Time PCR Detection System (Bio-Rad, Sundbyberg, Sweden). Hydrolysis probes were designed to bind over a splicing site to avoid amplification of potential genomic DNA contamination. The concentrations used for all primers were 900 nM and for all probes, 250 nM. All samples were analyzed in duplicate with ELF1α as reference gene based on previous work on reference gene stability [38]. In the distal intestine the reference gene showed lower expression in the +IPNV+F group compared to the +IPNV-F group (average Ct ± 1) but not to -IPNV. Therefore, a second reference gene, 18S rRNA, was tested. This reference gene has previously been shown to be stable in the intestine during viral challenge experiments [39] and proposed to be a good reference gene in tissues with high cell turnover [40], [41]. However, also the 18S gene showed a lower expression level in the distal intestine of the +IPNV+F group. For normalization of the mRNA expression of target genes in the distal intestine a geometric average of the two reference genes was used [42]. TaqMan qPCR was performed using commercial qPCR Master Mix No ROX Kit (Eurogentec, Liège, Belgium) in 20 µL reactions. The relative expression was calculated using the cycle threshold (Ct) number and the slope of the efficiency curve (E) of the reference gene and the target gene in the calculation:
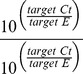
Primers for RNA expression of VP1, the RNA polymerase of IPNV, were previously published in Gadan et al 2012 [43] and used in 1000 nM concentrations for SYBRGreen detection of amplification after verification on the iQ5 platform, using the iQ SYBRGreen Supermix commercial kit in 20 µL reactions (Bio-Rad, Sundbyberg, Sweden). ELF1α was used as reference gene and melting curve analysis was used to verify specific products. Samples showing Ct<35 was considered positive for IPNV and are presented as filled circles in the mRNA expression figures.

**Table 1:** Primers and probes used for qPCR.

Gene		Sequence	Accession number	Ref
ELF α1	Forward	GCCCCTCCAGGACGTTTA	AF321836	[48]
	Reverse	CGGCCCACAGGTACAGTT		
	Probe	CCAATACCACCGATTTTG		
IFN type I	Forward	ACTGAAACGCTACTTCAAGAAGTTGA	AY216595, AY216594	[48]
	Reverse	GCAGATGACGTTTTGTCTCTTTCCT		
	Probe	CTGTGCACTGTAGTTCATTT		
Mx1 and 2	Forward	CCACCCTACAGGAGATGATGCT	U66475, U66476	[20]
	Reverse	ATCTGATCAGCCAAACGTTGACT		
	Probe	CTCAAGTCCTATTACAGGATATC		
CD3γδ	Forward	TGATCACCGGCAAGAAAACATCT	EF421418, EF421419	[70]
	Reverse	TTTGCACTCGCCTCATTGC		
	Probe	ATGAGCCCCATTTTGCT		
CD8α	Forward	AGAACGAAACGATGCCCACAT	AY693391, Y693393, AY701521	[71]
	Reverse	CTGTTGTTGGCTATAGGATGTTGTTG		
	Probe	TTCTCGGCTGTCTTTTG		
MHC-I	Forward	GCGACAGGTTTCTACCCCAGT	AF504013-25	[72]
	Reverse	TGTCAGGTGGGAGCTTTTCTG		
	Probe	TGGTGTCCTGGCAGAAAGACGG		
IL-1β	Forward	GCTCAACTTCTTGCTGGAGAGT	AY617117	[48]
	Reverse	GGGCGCCGACTCCAA		
	Probe	CTCCAACACTATATGTTCTTCC		
IFNγ	Forward	GCTGTTCAACGGAAAACCTGTTT	AJ841811, AY795563	[48]
	Reverse	GTCCAGAACCACACTCATCCA		
	Probe	TCACTGTCCTCAAACGTG		
IL-10	Forward	CCGTTTGACATCAAC GAGTTCATCT	AB118099	[73]
	Reverse	CAGCTCTCCCATTGCCTTATACAG		
	Probe	CCTTATCCTGCATCTTCT		
18S	Forward	CCCCGTAATTGGAATGAGTACACTTT	AJ427629	[39]
	Reverse	ACGCTATTGGAGCTGGAATTACC		
	Probe	CTTTAACGAGGATCCATTGG		
VP1	Forward	CTGGTCCAGAAACCCTAAGAC	AY379740	[43]
	Reverse	GTGTGTATCTCTCCCCTTTTGG		

The table shows the primers and hydrolysis probes (FAM-MGB NFQ probes; Applied Biosystems/Life Technologies Ltd, Paisley, UK) used for qPCR analyses of mRNA expression. The primers used for SYBRGreen detection of the IPNV polymerase VP1 is shown in the last row.

### Immunohistochemistry

The presence of CD8α, MHC-I and Mx was assessed by immunohistochemistry (IHC). For CD8α, a mouse monoclonal CD8α antibody, validated by Hetland et al [44], was used. For MHC-I a mouse monoclonal antibody was generated against the recombinantly expressed MHC-I heavy chain α 3 domain from Atlantic salmon. The reactivity of this antibody was validated by western blot on salmon red blood cells and by immunoprecipitation on biosynthetic labeled salmon spleen cells. For Mx protein expression, a polyclonal rabbit Ab directed against Atlantic salmon Mx was used [45]. At the time of analysis, tissue sections were dehydrated through an alcohol gradient, Histolab-clear (Histolab products, Västra Frölunda, Sweden) and imbedded in paraffin wax. Sections (6 µm) were sliced from proximal and distal intestines and mounted on 3'-aminopropyltriethoxysilane (APES; Sigma-Aldrich Sweden AB, Stockholm, Sweden) coated slides, dried at 37 °C for 24 h. Then the slides were dewaxed in Histolab-clear followed by rehydration in a series of alcohol baths followed by 1×10 min in ddH_2_O. The slides were then soaked in Tris buffered saline (6.06 g l^-1^ Tris HCl, 1.39 g l^-1^ Tris base, 9 g l^-1^ NaCl; pH 7.6) supplemented with 0.05% Tween-20 (TBST) 2×5 min. For two of the antibodies, mouse monoclonal CD8α and MHC-I, sections were subjected to epitope retrieval in Tris/EDTA (10/1 mM; pH9) containing 0.05% Tween-20 for 30 min at 100°C. Endogenous peroxidase activity was blocked by immersion in 0.3% H_2_O_2_ (in TBST:Methanol 7∶3) for 30 min at room temperature (RT) and then washed in TBST, 3×3 min. The slides were incubated with blocking solution (3% BSA, 5% normal goat serum and 5% normal donkey serum) in TBST for 1 h at RT. Excess blocking solution was removed and sections were incubated with 100 µl primary antibody diluted in blocking solution (CD8α; 1∶200, MHC-I alpha 3; 1∶600 and Mx 1∶4000) overnight in a humid box at 4 °C. Blocking solution without primary antibody served as controls. Thereafter, all incubations were performed at RT. The slides were washed in TBST 3×3 min and incubated with a biotinylated secondary Ab for 1 h. Thereafter the slides were washed in phosphate buffered saline (PBS; pH 7.2) with 0.05% Tween-20 (PBST) 3×3 min and incubated with 100 µl Vectastain Elite ABC reagent (according to Vector kit instructions) for 30 min followed by NovaRED peroxidase substrate solution (according to Vector kit instructions) for 7 min, then washed again for 10 min in running tap water, dehydrated in alcohol gradient followed by Histolab-clear and mounted with Pertex (Histolab Products AB, Västra Frölunda, Sweden). For assessment of the number of CD8α positive cells, pictures were taken using a Nikon Eclipse E1000 microscope equipped with a Nikon DXM 1200 digital camera. The pictures were imported into Biopix iQ software (Bio Pix AB, Gothenburg, Sweden) where the number of cells in the intestinal epithelium divided by the length of epithelia observed were determined. The immune staining of MHC-I and Mx was scored from 0-5 where 5 was the highest color intensity observed. For Mx, the scoring was divided between Mx expressed in the enterocytes and expression in other cell types such as in goblet cells, termed other.

### Statistics

The data was analyzed using a 1-factorial analysis of variance (ANOVA) followed by Tukeýs post hoc test. Homogeneity was tested using Levenés test and data not passing the test were transformed using log10 transformation. Heterogeneous data and semi-quantitative scores for MHC-I and Mx protein expression were subjected to Kruskal-Wallis non-parametric test followed by Bonferroni corrected Mann-Whitney U test to determine differences between groups. Statistical tests were performed using SPSS 18.0 (SPSS Inc., Chicago, Illinois). All data were expressed as means±SEM and *p*<0.05 was regarded as significant. The difference in survival rate was assessed using a Kaplan Meier approach [46].

## Results

**Figure 1:**
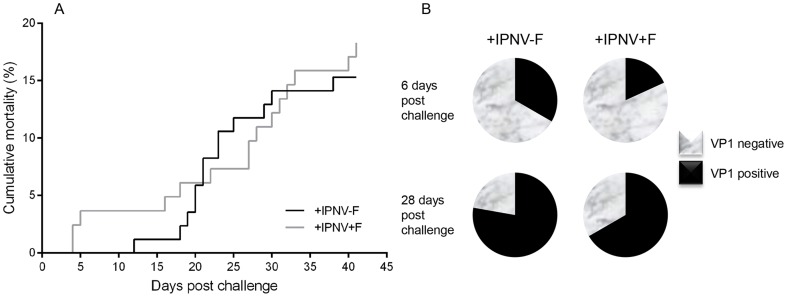
Mortality and IPNV prevalence following IPNV cohabitant challenge and cortisol treatment. Cumulative mortality during the experimental period in the +IPNV-F group and the +IPNV+F group (A) and the IPNV prevalence as analyzed by VP1 RNA expression 6 and 28 days post-challenge (B). 6 days post-challenge VP1 was detected in 33% and 18% of the examined fish from the +IPNV-F and +IPNV+F groups respectively (total average 26%). 28 days post-challenge 78% and 67% of the fish was tested positive from the +IPNV-F and +IPNV+F groups respectively (total average 72%).

The cumulative mortality curves were sigmoidal in shape for both groups and no statistical difference was found between the two treatments. However, mortality started in the +IPNV+F group at day 4 post-challenge (i.e. introduction of cohabitant fish; Fig. 1A) whereas it did not start until day 12 in the +IPNV-F group and the slope appeared steeper in this group. Viral RNA coding for VP1 was found in 26% of the fish analyzed at day 6 (33% in the +IPNV-F and 18% in the +IPNV+F group) and in72% at day 28 post-challenge (78% in the +IPNV-F and 67% in the +IPNV+F group; Fig. 1B). Further, there seems to be a higher expression of the viral protein in the head kidney compared to the proximal intestine after 28 days of cohabitant challenge as suggested by the higher relative expression values in the head kidney (Fig. S1).

**Figure 2:**
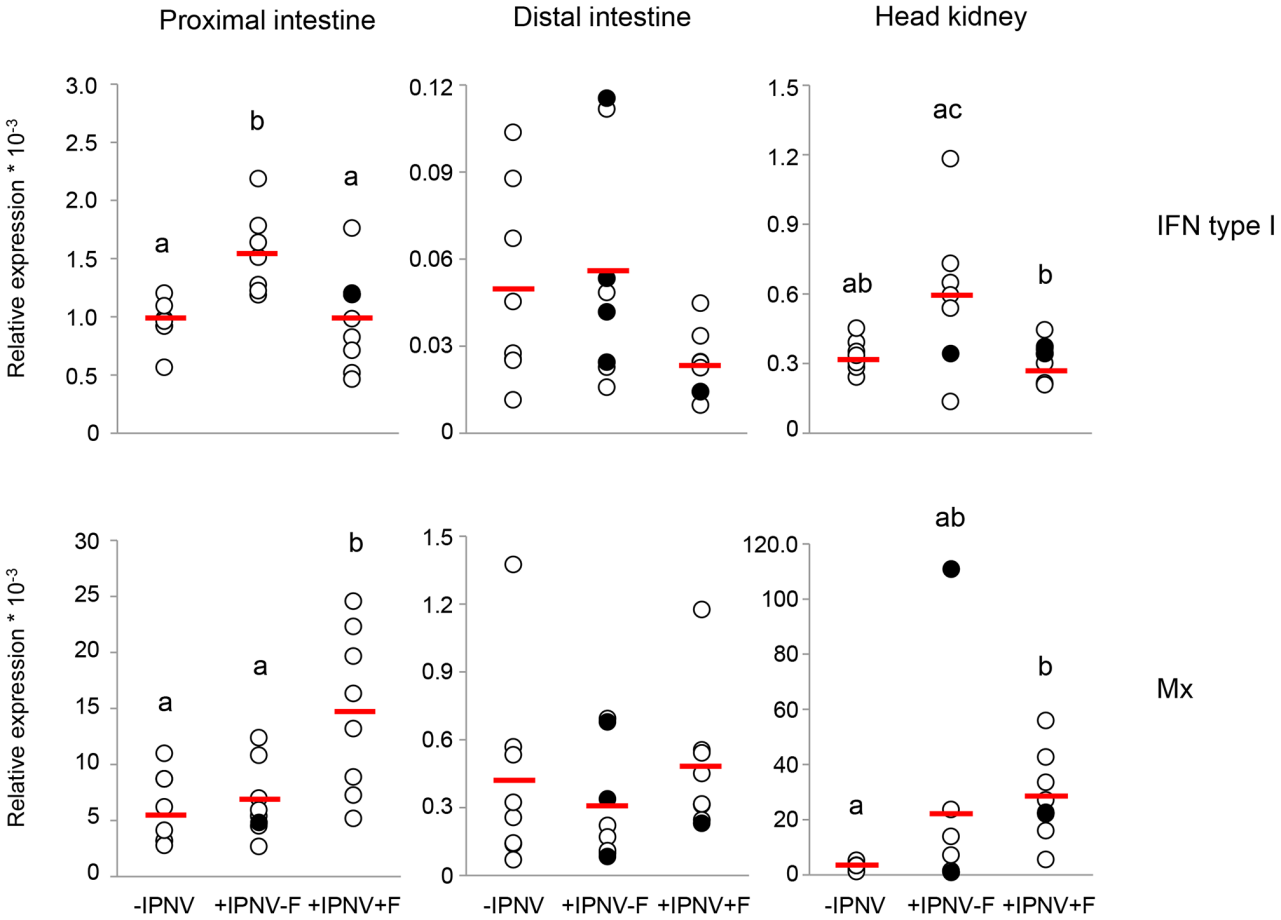
mRNA expression of IFN type I and Mx 6 days post-challenge. mRNA expression of IFN type I in the proximal intestine (n = 7, 7, 9), distal intestine (n = 8, 8, 7) and in the head kidney (n = 7, 7, 8) and mRNA expression of Mx in the proximal intestine (n = 8), distal intestine (n = 8) and in the head kidney (n = 8, 7, 8). The VP1 positive fish 6 days post-challenge is presented as filled circles and the red line corresponds to the average value of the treatment group. Different letters denote statistically significant differences among the groups according to Tukeýs post hoc test. For Mx, Kruskal-Wallis non-parametric test was used followed by Bonferroni corrected Mann-Whitney U test to determine differences between groups. Outliers with values outside average ± 2 x SD were omitted. Statistical significance was accepted at p<0.05.

**Figure 3:**
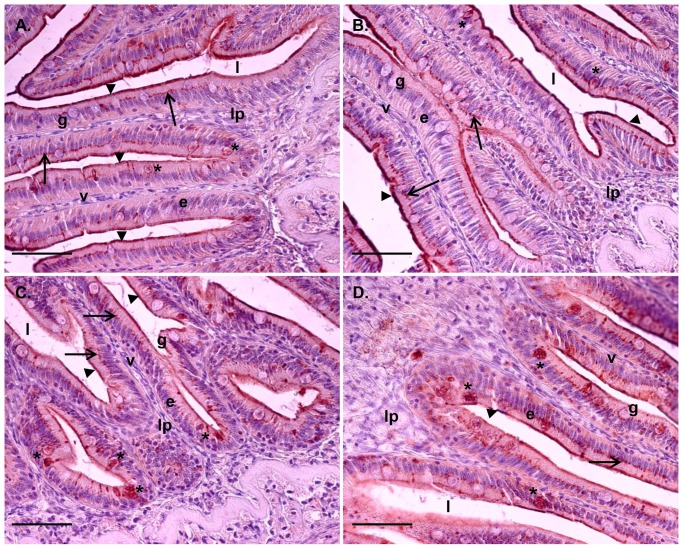
Tissue localization of Mx immune staining in the proximal and distal intestine. Tissue sections, after 6 days of cohabitant challenge, from proximal intestine (A and B) and distal intestine (C and D). Scale bar 50 µm. Epithelial cells (e) lining the villi (v) show supra nuclear presence of Mx (arrow). Mx is also present in the most apical part of the enterocytes in the micro villi region (arrowhead). Staining could also be found in goblet cells (g) (*). The lamina propria (lp) and intestinal lumen (l) are featured for clarity.

Six days of IPNV challenge resulted in a significantly higher IFN type I mRNA expression in the proximal intestine in +IPNV-F treated fish compared to the -IPNV group. Further, the proximal intestine and head kidney of +IPNV-F treated fish showed higher mRNA expression of IFN type I compared to the +IPNV+F group (Fig. 2). The cortisol implant in the +IPNV+F group abolished the effect of the immune challenge on mRNA expression of IFN type I, as this did not raise above background level (-IPNV) after implantation with cortisol. In the proximal intestine, the mRNA expression of Mx was higher in the +IPNV+F group compared to the +IPNV-F and the –IPNV, while in the head kidney the Mx expression was higher in the +IPNV+F group compared to the –IPNV group (Fig. 2). This was supported by a higher staining intensity for Mx in the enterocytes of the proximal intestine in the +IPNV+F group as compared to the -IPNV (Kruskal-Wallis p<0.05; Table 2). No significant differences were observed in the distal intestine (Fig. 2). In general, Mx staining was observed in a supra nuclear position of enterocytes as well as in the most apical part of the enterocytes in the microvilli region (Fig. 3). Positive staining could also be observed in other cell types such as goblet cells and cells in the *lamina propria* (Fig. 3).

**Table 2:** Mx immune staining 6 days post-challenge.

	Mean score of Mx in the intestine					
	-IPNV (n = 3)		+IPNV-F (n = 6)		+IPNV+F (n = 5)	
	Mean	SEM	Mean	SEM	Mean	SEM
Proximal intestine						
Enterocyte (p<0.05)	3.6^a^	0.22	3.9^ab^	0.35	4.7^b^	0.12
Other	2.0	0.26	3.0	0.27	2.2	0.29
Distal intestine						
Enterocyte	2.9	0.22	2.7	0.20	2.8	0.27
Other	2.8	0.17	3.4	0.23	2.9	0.42

The Mx immune reactivity in the proximal and distal intestine was scored 0-5 with 5 being the most abundant reactivity (n = 3-6). The expression in enterocytes was separated from the expression in other cell types such as goblet cells and leucocytes, termed other. Statistical difference was accepted at p<0.05 as determined by Kruskal-Wallis non-parametric test followed by Bonferroni corrected Mann-Whitney U test to determine differences between groups.

**Figure 4:**
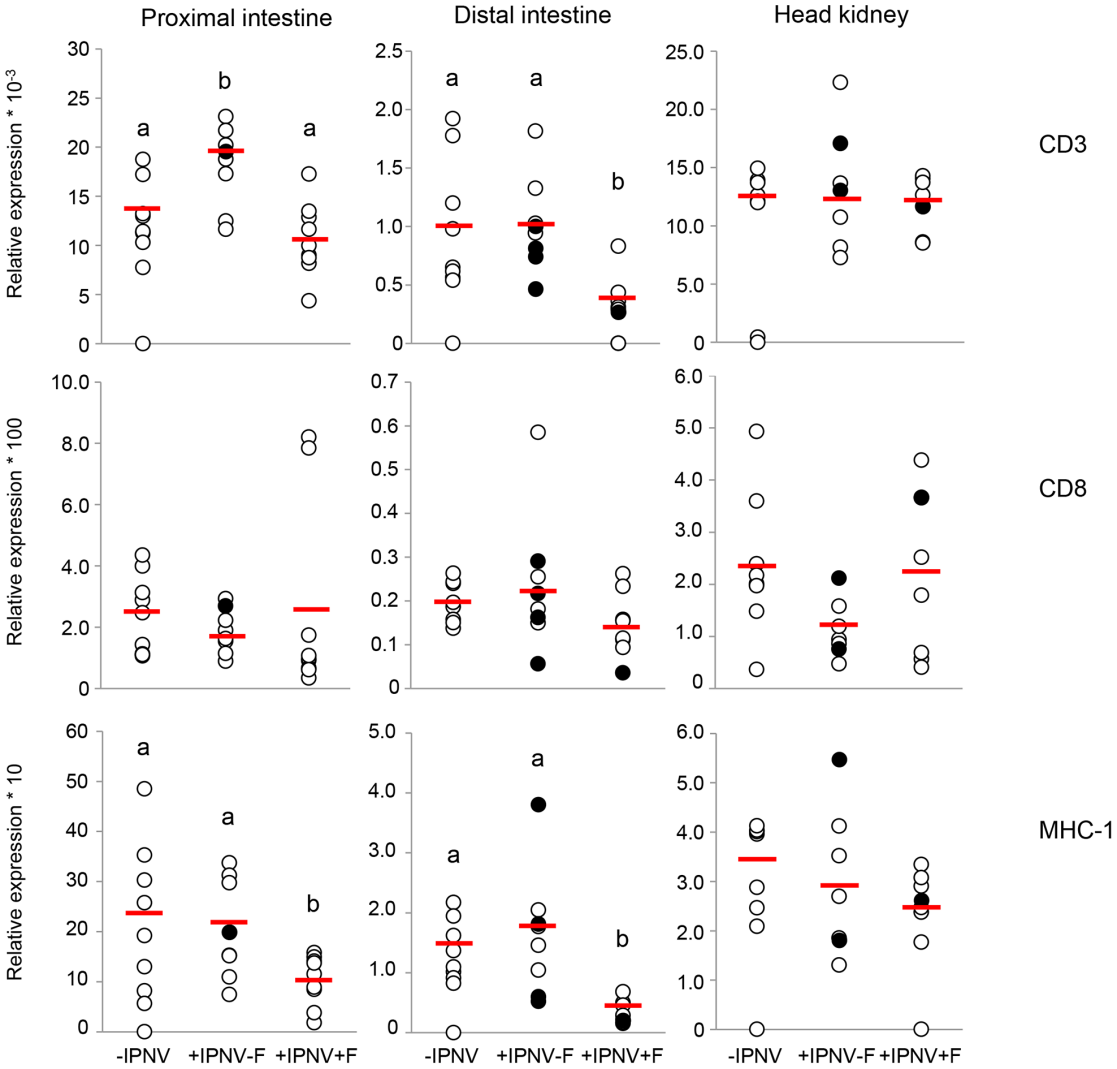
mRNA expression of CD3, CD8α and MHC-I 6 days post-challenge. mRNA expression of CD3 in the proximal intestine (n = 9, 8, 9), distal intestine (n = 9, 8, 8) and in the head kidney (n = 8, 7, 8), mRNA expression of CD8α in the proximal intestine (n = 9, 8, 9), distal intestine (n = 8) and in the head kidney (n = 8, 7, 8) and mRNA expression of MHC-I in the proximal intestine (n = 9, 8, 9), distal intestine (n = 9, 8, 8) and in the head kidney (n = 8, 7, 8). The VP1 positive fish 6 days post-challenge is presented as filled circles and the red line corresponds to the average value of the treatment group. Different letters denote statistically significant differences among the groups according to Tukeýs post hoc test. Statistical significance was accepted at p<0.05.

**Figure 5:**
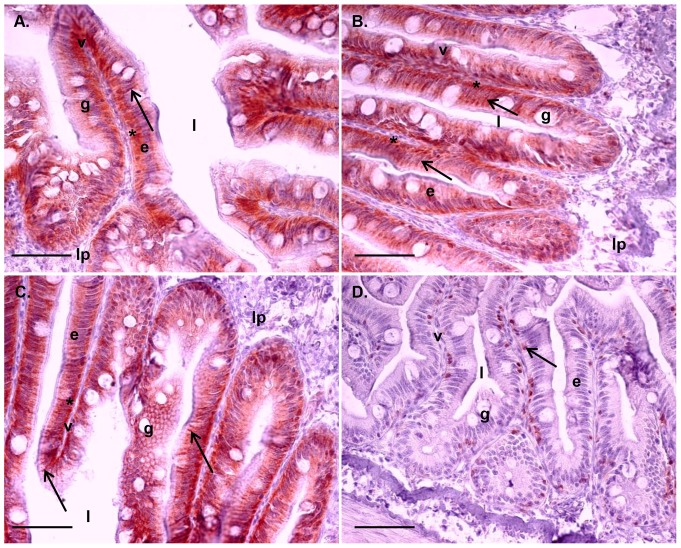
Tissue localization of MHC-I and CD8α immune staining in the proximal and distal intestine. Tissue sections, after 6 days of cohabitant challenge, showing presence of MHC-I in the proximal intestine (A and B) and distal intestine (C). Scale bar 50 µm. Epithelial cells (e) lining the villi (v) show staining of MHC-I in the basal (*) and lateral region (arrowhead) of the epithelial cells. MHC-I is also observed as a band in the supra nuclear region of the enterocytes (arrow). Interspersed goblet cells (g) and lamina propria (lp) and luminal space (l) featured for clarity. Presence of CD8α in the proximal intestine is shown lining the space between the epithelial cell layer and the lamina propria (arrow; D).

CD3 mRNA expression was significantly higher in the proximal intestine after 6 days of IPNV challenge (+IPNV-F group) compared to the -IPNV group and the +IPNV+F group (Fig. 4). The +IPNV+F group had significantly lower CD3 mRNA expression in the distal intestine than the -IPNV group and the +IPNV-F group (Fig. 4). No significant effect was seen in the head kidney (Fig. 4). Neither 6 days of IPNV challenge nor cortisol treatment resulted in any significant effects in CD8α mRNA expression (Fig. 4). However, IHC revealed a significantly lower number of CD8α expressing cells in the proximal intestine of the +IPNV+F group compared to the -IPNV (Table 3). In both intestinal segments the MHC-I mRNA expression was significantly lower in the +IPNV+F group compared to the -IPNV group (Fig. 4). In the distal intestine, the mRNA expression in the +IPNV+F group was further significantly different also when compared to the +IPNV-F group (Fig. 4). The MHC-I immune reactivity was abundant on the enterocytes, located to the basolateral and basal sides and in close connection to the CD8α immune reactive cells which were mainly present near the basal ends of the enterocytes (Fig. 5 A-D). No significant differences in MHC-I immune staining between the treatment groups could be found using IHC analyzes (Table 4).

**Table 3:** CD8α immune staining 6 days post-challenge.

	Number of CD8α+ lymphocytes * mm^-1^ epithelia					
	-IPNV (n = 3)		+IPNV-F (n = 6)		+IPNV+F (n = 6)	
	Mean	SEM	Mean	SEM	Mean	SEM
Proximal intestine (p<0.05)	32.0^a^	7.0	21.1^ab^	2.9	15.1^b^	1.3
Distal intestine	36.2	6.3	35.8	4.6	32.9	2.6

The number of CD8α expressing lymphocytes presented as number of cells in the proximal and distal intestinal epithelium divided by the length of epithelia observed (n = 3-6). Different letters indicate statistical differences after Tukeýs post-hoc test. Statistical difference was accepted at p<0.05.

**Table 4:** MHC-I immune staining 6 days post-challenge.

	Mean score of MHC-I in the intestine					
	-IPNV (n = 3)		+IPNV-F (n = 6)		+IPNV+F (n = 5)	
	Mean	SEM	Mean	SEM	Mean	SEM
Proximal intestine	4.6	0.19	4.1	0.18	3.8	0.35
Distal intestine	3.9	0.08	3.7	0.14	4.1	0.26

The MHC-I immune staining in the proximal and distal intestine was scored 0-5 with 5 being the most abundant staining (n = 3-6). Statistical difference was accepted at p<0.05.

**Figure 6:**
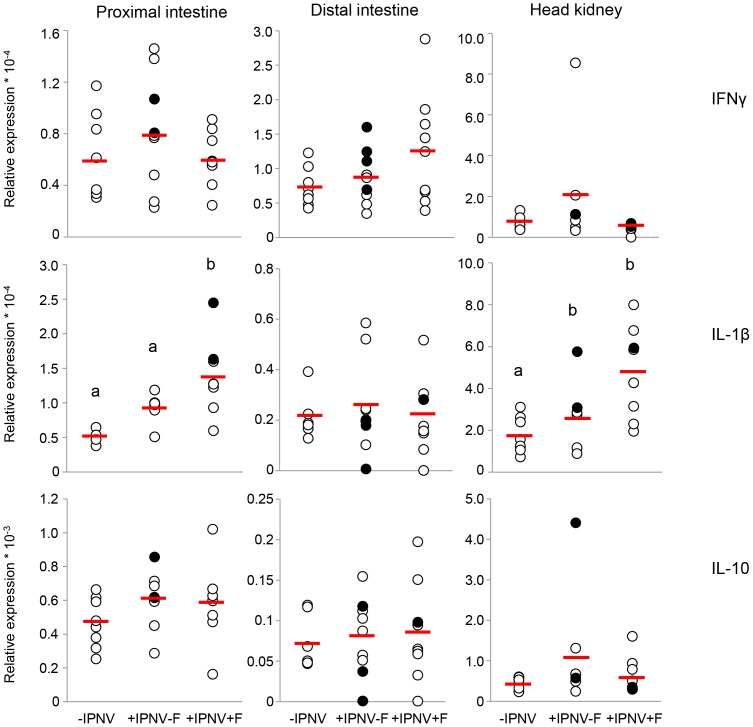
mRNA expression of IL-1β, IFNγ and IL-10 6 days post-challenge. mRNA expression of IL-1β in the proximal intestine (n = 4, 7, 8), distal intestine (n = 7, 9, 9) and in the head kidney (n = 8, 7, 8), mRNA expression of IFNγ in the proximal intestine (n = 8, 9, 8), distal intestine (n = 8, 9, 9) and in the head kidney (n = 7, 7, 8) and mRNA expression of IL-10 in the proximal intestine (n = 8, 9, 8), distal intestine (n = 7, 9, 9) and in the head kidney (n = 8, 7, 8). The VP1 positive fish 6 days post-challenge is presented as filled circles and the red line corresponds to the average value of the treatment group. Different letters denote statistically significant differences among the groups according to Tukeýs post hoc test. Statistical significance was accepted at p<0.05.

IL-1β mRNA expression was elevated in the proximal intestine in cortisol implanted infected fish compared to the –IPNV group, whereas no effect was seen by IPNV alone (Fig. 6). There was further a significantly higher mRNA expression in the head kidney +IPNV-F group and the +IPNV+F group compared to the -IPNV group (Fig. 6). No significant effect was seen in the distal intestine (Fig. 6). The treatments did not affect IFNγ or IL-10 mRNA expression 6 days post-challenge (Fig. 6).

## Discussion

In the current study, cortisol implants resulted in elevated plasma cortisol levels within the range observed during a stress response [31]. Further, fish were successfully infected with IPNV through natural infection routes created by a cohabitant challenge. IPNV infection was verified both after 6 and 28 days of the challenge, as observed by the presence of viral VP1 transcripts in 26% and 72% of the examined fish, respectively. Further, after 28 days the RNA expression in the head kidney was higher than in the intestine in both infected groups suggesting a viral manifestation in the tissue. The head kidney is routinely used in IPNV diagnostics and the presence of virus in the kidney is normally detected within a week in infected fish [47], [48]. Since viral mRNA was found also in the intestine (Figs. 2, 4 and 6), the results verifies this as a likely route of infection and the head kidney as a viral target.

The higher mRNA expression of IFN type I, found in the +IPNV-F group compared to both –IPNV and +IPNV+F fish, in the proximal intestine and the head kidney is indicative of the early innate anti-viral response [43]. The simulated chronic stress in terms of elevated plasma cortisol levels, suppresses the response both systemically and locally in the intestine. Accordingly, Atlantic salmon fry infected with IPNV and subjected to repeated stress for 7 days showed a suppressed expression of IFN type I mRNA [35]. Reduced IFN type I mRNA expression of *in vitro* isolated head kidney macrophages was also observed when Atlantic salmon post smolts were subjected to chronic husbandry stress and immune challenge by poly I:C treatment [20].

One of the mechanisms of the IFN type I pathway to inhibit viral replication is through up-regulation of Mx [24]. In the +IPNV+F group there is an increased mRNA as well as protein expression of Mx in the proximal intestine and an increased Mx mRNA expression in the head kidney without up-regulation of IFN type I. The observed divergences in IFN type I and Mx mRNA expression could be explained by a difference in timing between treatment groups as well as individuals. For example, a sampling capturing an early point in IFN type I pathway activation may show an increased IFN type I mRNA expression without any increased expression in Mx mRNA. Along this line, the Mx mRNA expression in the +IPNV+F group may be activated by stimulatory action by IFN type I that have returned to basal levels at day 6. However, the discrepancies can also reflect an anti-viral response induced by other cytokines than IFN type I. In this respect, IL-1β and IFNγ has been suggested as possible Mx inducers [49], [50]. For example, the functional P1 domain of IL-1β was shown to induce Mx expression in rainbow trout after intra-peritoneal injection [50]. In the present study, IL-1β but not IFNγ mRNA is up-regulated in both proximal intestine and head kidney in support of such regulatory effect by IL-1β. Furthermore, an overall immunosuppressive effect of cortisol could result in increased virus replication, which in turn would increase the Mx expression [20], [35]. In the infected, vehicle implanted fish, on the other hand, there is no increased Mx mRNA expression despite an increased mRNA expression of IFN type I. This could suggest an IPNV dependent inhibition of the Mx mRNA expression at the time of sampling and hence a suppression of the anti-viral response at the level of Mx. There have been reports of a balance between the IPNV infection process and the IFN type I/Mx activation process [51]. Cells pre-treated with recombinant IFN type I suppressed IPNV proliferation while the anti-viral response was inhibited at the Mx promoter region level when the cells were already infected [51].

The enterocyte distribution of Mx, being supra nuclear as well as close to or in the apical membrane is intriguing. In a majority of vertebrate species, two or more different genes of Mx have been characterized and different protein isoforms with different functions have been suggested [52]. Presence of Mx in the cytoplasm close to the nucleus is believed to have a protective role against viral replication [24], whereas a more apical localisation of Mx and presence in mucus secreting cells may indicate the possibility of a secretory form of Mx [53], [54]. Ovine Mx1 is secreted with exosomes from uterus epithelial cells [53] and suggested to regulate unconventional vesicular secretory processes [54]. The localization of Mx in the most apical region of the enterocytes as well as in the goblet cells, as shown in the present study, resembles previous observations also in the gills of Atlantic salmon [45]. Thus, the possibility of several Mx isoforms as well as secretory forms, equivalent to the mammalian Mx1, in salmonids merits further investigation.

CD3 is expressed on all T cells as a part of the T cell receptor complex and is involved in signal transduction upon receptor activation [55]. The increased CD3 mRNA expression in the proximal intestine of the +IPNV-F group compared to the non-challenged group, indicate that T-cells are important in this intestinal region during an IPNV infection. The increased CD3 mRNA expression is not accompanied by increases in CD8α or MHC-I mRNA expression in this intestinal region and treatment group. This may indicate an early T cell proliferation and a differentiation of T cells, e.g. T_regs_ or T_h_ cells, towards CD4 expression rather than CD8 [25]. The absence of effect on the CD8/MHC-I system by the IPNV challenge, could also be an effect of sampling time, *i.e*. that this part of the intestinal mucosal immune system still show basal expression levels at day 6 post-challenge. In both intestinal regions the mRNA expression of CD3 is significantly lowered by the cortisol treatment in the IPNV challenged fish (+IPNV+F). This suggests an immunosuppressive effect of the stress hormone on T-cells [56]. In concordance with the results on CD3 mRNA expression, the MHC-I mRNA expression as well as the number of CD8α lymphocytes are decreased in response to cortisol. These results give further support to an immunosuppressive effect of cortisol on the response mediated by cytotoxic T-cells. The intestinal epithelial immune system is constantly exposed to a large variety of antigens, which probably results in high constitutive expression levels as indicated by the ability of cortisol to suppress these markers. In mammals, MHC-I and II are present on intestinal epithelial cells (IECs) and increases in abundance as a response to infection as well as stress [57]. IECs act as antigen sampling and antigen presenting cells and activate CD8α positive cells [58]. The overall higher expression of CD8α protein in the distal intestine compared to the proximal intestine could therefore be an evidence for a higher level of antigen sampling and presentation in this area.

A cohabitant challenge results in viral exposure through the primary barriers. In fish, these are the skin, gills and the gastrointestinal tract, with the intestine positively demonstrated as an entrance route for IPNV [32]. Through the ingested water, the proximal intestine becomes exposed to the viral particles first and there is probably an initially higher load of virus in this segment compared to the distal intestine. This is further indicated by assessment of the intestinal permeability after viral infection, which was shown to be more affected in the proximal compared to the distal intestine [32]. Furthermore, binding of virus to mucus secreted along the intestine may result in the creation of a mucus tube with trapped virus particles in the intestinal lumen, leading to a lesser degree of viral exposure further down in the intestine [59], [60]. Therefore, the proximal intestine is a likely target tissue for the initiation of IPNV infection. In this study, the effect on immune response markers is similarly more evident in the proximal intestine compared to the distal intestine after 6 days of cohabitant challenge suggesting an earlier and/or greater exposure to the virus in the proximal intestine. Accordingly, previous studies on salmonids demonstrates more pronounced effects in the proximal compared to the distal intestine in terms of visible signs of infection as well as immune response against the virus [30], [31],[61].

A distinguished feature of the response towards many virus, including IPNV, is an increase in IFN type I expression and the presented results show a significant increase in both the head kidney and proximal intestine in the +IPNV-F group 6 days post-challenge. Concomitantly, the mortality curves indicate an initially lower mortality in the +IPNV-F group compared to the +IPNV+F group despite more VP1 positive individuals detected in this group. Similarly, McBeath et al [48] showed lower level of viral transcripts in non-injected fish compared to injected cohabitants and this was concomitant with a higher mortality rate. Hence, there is no clear correlation between high levels of viral transcripts and mortality, which may be a result of an increased anti-viral defense through increased expression of IFN type I.

Variations in expression of IFN type I as well as in other markers was found. IPNV infections can result in high mortalities in susceptible individuals but also in persistent virus carrying cells in surviving fish. It is therefore likely that the response differs depending on the predisposition of an individual to the latent or the lethal form of IPN disease [62]. In the +IPNV-F group, fish expressing RNA for the polymerase VP1 generally also expressed higher or lower than average mRNA levels of anti-viral markers IFN type I, IFNγ and Mx (Fig. 2 and 6). The overall picture suggests a high mRNA expression of anti-viral markers in the VP1 positive fish in response to IPNV infection and a suppression of this response in the presence of cortisol. Further, IL-1β mRNA expression was increased in the head kidney of both infected groups with VP1 positive fish showing expression above the group average. This is in line with previous findings and could potentiate the hypothesis that the large amounts of macrophages present in the head kidney give rise to increased levels of the cytokine in infected fish [63]. A time dependent increase in IL-10 mRNA expression has been observed in the head kidney of IPNV infected persistent fish and this immune modulator may account for a decreased mortality rate in infected fish through a dampening of the inflammatory processes [63]. In the present study, some VP1 positive fish in the +IPNV-F group showed a high mRNA expression of IL-10 while others were at intermediate expression level.

In a cohabitant challenge with IPNV, mortality is expected in the injected cohabitants after 1-2 weeks while disease and increase in mortality in the non-injected fish is expected after 2-4 weeks [48], [64], [65]. In the vehicle implanted group, mortality of non-injected fish started at day 12 post-challenge, a time of onset well in line with previous studies, whereas there was an earlier onset of mortality (day 4) in the cortisol implanted group. A similar early mortality onset (day 3, post-IPNV challenge) has been seen shown in cohabitantly challenged Atlantic salmon smolts exposed to environmental stress [66]. Furthermore, the time for onset of mortality can be stock density dependent and fish densities corresponding to the densities used in the current study (over 16 kg m^-3^) has previously resulted in mortalities as early as day 3 post-IPNV challenge [67]. The early onset in mortality in the cortisol implanted group could therefore be a result of the density of fish in the tanks together with the cortisol treatment, mimicking a stress response. The cumulative mortality at the end of the present experiment reached approximately 20% in both groups, which is within the range of previously reported mortalities for cohabitant challenges.

The mortality data thus suggest that cortisol enhance the onset of mortality in IPNV cohabitants, with a steady increase in mortality in the +IPNV+F group up to day 41 at which point the mortality in the +IPNV-F group have risen to approximately the same level. This is well in line with the demonstrated suppressive effect of cortisol on several of the immune parameters assessed, especially in the intestinal epithelial immune system. The results further indicates that the infection and hence immune response is activated but in an early state day 6 post-challenge.

## Conclusions

IPN disease outbreak depends on an intricate balance between the innate and acquired immune response of the host, cell signaling and internal pathways and virus induced alteration of immune function [68], [69]. The current study show an up-regulation of the anti-viral cytokine IFN type I in the proximal intestine with a subsequent suppression of this cytokine by cortisol. There is further a down-regulation of the T cell marker CD3 and MHC-I in response to cortisol treatment. In terms of general response towards IPNV, there is a clear induction of immune markers in the proximal intestine whereas the distal intestine is affected by cortisol but show weaker response towards the pathogen. The head kidney mRNA expression data corresponds to the proximal intestine in terms of the anti-viral response and an increase is seen in the pro-inflammatory cytokine IL-1β in response to IPNV infection. There is however a lesser effect on the cellular immune response in the head kidney.

Therefore, the present study clearly demonstrate differentiated epithelial immune responses in different regions of the intestine as well as a local immune response different from the systemic, in response to IPNV infection as well as to cortisol treatment in Atlantic salmon.

## Supporting Information

Figure S1 **RNA expression of VP1 in the head kidney and intestine 28 days post-challenge.** RNA expression of VP1 in the head kidney (n = 4, 5) and in the proximal intestine (n = 6, 5). No significant difference was found between treatments 28 days post challenge. * denote statistically significant difference between tissues according to Tukeýs post hoc test. Statistical significance was accepted at p<0.05.
(TIF)
