# Supplementary figures and images for: Effects of Cortisol on the Intestinal Mucosal Immune Response during Cohabitant Challenge with IPNV in Atlantic Salmon (Salmo salar)
Source: PLoS One. 2014 May 8;9(5):e94288. doi: 10.1371/journal.pone.0094288 (PMC4014467; doi:10.1371/journal.pone.0094288)

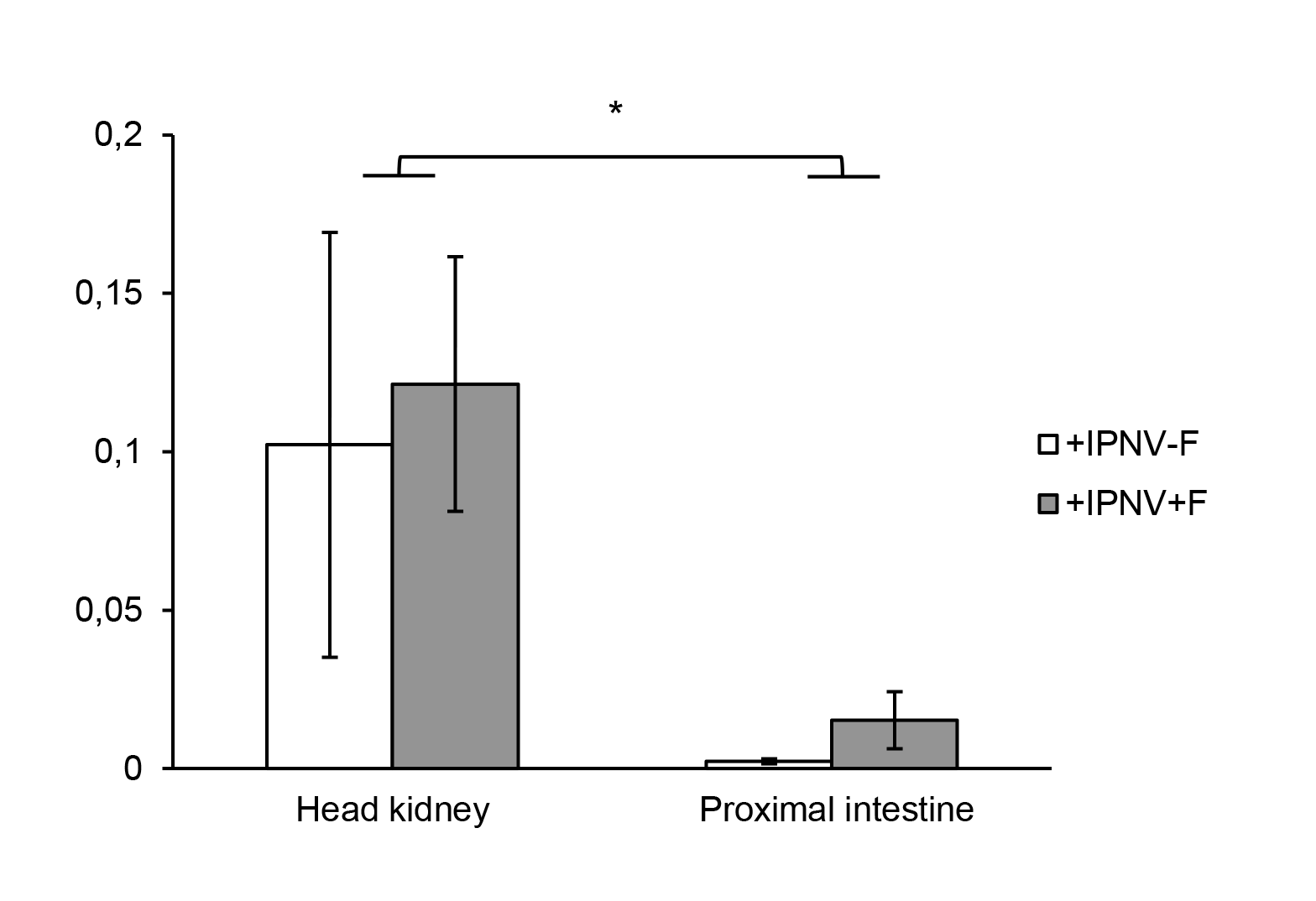

Supplement: Figure S1 — RNA expression of VP1 in the head kidney and intestine 28 days post-challenge. RNA expression of VP1 in the head kidney (n = 4, 5) and in the proximal intestine (n = 6, 5). No significant difference was found between treatments 28 days post challenge. * denote statistically significant difference between tissues according to Tukeýs post hoc test. Statistical significance was accepted at p<0.05. (TIF) [file pone.0094288.s001.tif]
